# Serial blood serum measurements of calprotectin and deoxyribonuclease in COVID-19 patients during hospitalization and recovery until one year: a prospective, multicenter, observational study

**DOI:** 10.1186/s12879-026-12939-x

**Published:** 2026-03-02

**Authors:** Anders Benjamin Kildal, Thor Ueland, Mohammad Reza Mirlashari, Lise Sofie Nissen-Meyer, Beathe Kiland Granerud, Anne Ma Dyrhol-Riise, Kristian Tonby, Aleksander Rygh Holten, Lars Heggelund, Karl Erik Müller, Anders Tveita, Simen Bøe, Magne Kristoffer Fagerhol, Geir Hetland, Jan Cato Holter

**Affiliations:** 1 Anesthesia and Critical Care Research Group, Department of Clinical Medicine, Faculty of Health Sciences, UIT – The Arctic University of Norway, Tromsø, 9019 Norway; 2 Department of Anesthesiology and Intensive Care, University Hospital of North Norway, Tromsø, 9019 Norway; 3 Research Institute of Internal Medicine, Oslo University Hospital, Oslo, 0424 Norway; 4 Institute of Clinical Medicine, University of Oslo, Oslo, 0315 Norway; 5 Thrombosis Research Center (TREC), Division of internal medicine, University hospital of North Norway, Tromsø, 9019 Norway; 6 Department of Immunology and Transfusion Medicine, Oslo University Hospital Ullevål, Oslo, 0450 Norway; 7 Department of Microbiology, Oslo University Hospital, Oslo, 0424 Norway; 8 Department of Infectious Diseases, Oslo University Hospital, Oslo, 0424 Norway; 9 Department of Acute Medicine, Oslo University Hospital, Oslo, 0424 Norway; 10 Department of Internal Medicine, Drammen Hospital, Vestre Viken Hospital Trust, Drammen, 3004 Norway; 11 Department of Clinical Science, Faculty of Medicine, University of Bergen, Bergen, 5009 Norway; 12 Section of Clinical Immunology and Infectious Diseases, Oslo University Hospital, Oslo, 0424 Norway; 13 Department of Internal Medicine, Bærum Hospital, Vestre Viken Hospital Trust, Gjettum, 1346 Norway; 14 Department of Anesthesiology and Intensive Care, Hammerfest County Hospital, Hammerfest, 9601 Norway

**Keywords:** Calprotectin, S100A8/A9, Myeloid-related protein 8/14, Deoxyribonuclease, DNase, COVID-19, SARS-CoV-2

## Abstract

**Objectives:**

To examine circulating calprotectin and deoxyribonuclease (DNase) serum levels during hospitalization in COVID-19 patients, their continuity post-discharge and dynamics in relation to disease severity.

**Methods:**

Serum levels were measured in samples collected from 252 COVID-19 patients during hospitalization (admission, days 3–5 and 7–10), at 3 months and after 1 year, and related to admission to intensive care unit (ICU) or high dependency unit (HDU), and 60-day total mortality.

**Results:**

During hospitalization, calprotectin and DNase levels were significantly elevated compared to healthy controls (HC). Calprotectin was increased in ICU/HDU patients compared to those at wards during hospitalization and in non-survivors compared to survivors during prolonged hospitalization. High calprotectin levels at admission were associated with male sex, PaO2/FiO2 ratio and national COVID-19 wave 2 and 3 compared to wave 1. High admission levels of both calprotectin and ferritin were associated with an approximately twofold higher odds ratio for ICU/HDU admission than either marker alone. DNase was lower at admission in non-survivors compared to survivors. Post-discharge, DNase but not calprotectin levels remained elevated extending to 1-year follow-up compared to HC.

**Conclusions:**

COVID‑19 non‑survivors showed persistently higher calprotectin levels during prolonged hospitalization, and elevated admission calprotectin was associated with later pandemic waves and poorer oxygenation. Incorporating calprotectin with ferritin improved prediction of ICU/HDU admission. DNase levels were lower at admission in non‑survivors, while overall DNase remained elevated from hospitalization through 1‑year follow‑up compared with HC, suggesting a possible role in long‑term COVID‑19–related immune alterations.

**Supplementary Information:**

The online version contains supplementary material available at 10.1186/s12879-026-12939-x.

## Introduction

Circulating Calprotectin (S100A8/A9 or myeloid-related protein 8/14) and neutrophil extracellular traps (NETs) are neutrophil-derived inflammatory markers and part of the innate immune system. Both calprotectin and NETs have been linked to severity and mortality in hospitalized COVID-19 patients [1, 2] as a result of respiratory failure, sepsis and multiorgan failure [3]. Deoxyribonuclease (DNase) may disrupt NETs by digesting their DNA content, and low levels have been related to sepsis and death in hospitalized COVID-19 patients [4]. The exact role of calprotectin, NETs and DNase in disease progression remains to be fully understood.

Neutrophil contact with invading microorganisms makes the neutrophils either engulf them, release antimicrobial granules, or form NETs [5]. Calprotectin is attached to NETs contributing to the antimicrobial activity of NETs, but may lead to cell and tissue collateral damage [6]. Endogenous DNase is produced by the host itself, to recycle NET-structures and thus avoid tissue-damaging effects of NETs during infections or noninfectious diseases [7–9].

Only few studies [1, 10–14] have performed serial measurements of serum calprotectin in cohorts of hospitalized patients to assess this marker as a COVID-19 monitor, but not for an extended period up to one year after hospital admission and not in combination with DNase. Since these markers have opposing roles in the inflammatory response, a deeper understanding of the kinetics of both calprotectin and DNase during COVID-19 hospitalization and recovery post-discharge is needed. Hence, the aim of this study was to examine the dynamic changes of serum calprotectin and DNase levels during hospitalization in relation to disease severity and 60-day all-cause mortality as well as their temporal profiles at 3 and 12 months of follow-up in a well-characterized cohort of hospitalized COVID-19 patients.

## Material and methods

### Patients and clinical outcomes

Study participants were recruited from the Norwegian SARS-CoV-2 study (NCT04381819), a prospective multicenter cohort study of COVID-19 patients admitted to five Norwegian hospitals, conducted as part of an International Severe Acute Respiratory and Emerging Infection Consortium (ISARIC) WHO Clinical Characterization Protocol study, as previously described [15]. Participants aged ≥ 18 years and admitted to hospital with PCR-confirmed SARS-2-CoV-2 infection were eligible for inclusion and were included from March 2020 until September 2021. This period encompassed the first three COVID-19 waves in Norway, estimated as the following periods: wave one, 8 March 2020 to 31 July 2020; wave two, 1 August 2020 to 17 February 2021; and wave three, 18 February 2021 to 31 July 2021. From February 2021, Alpha was the dominating variant, replaced by the Delta variant from July 2021. Blood samples for biochemistry and biobanking were obtained within 48 h after hospital admission, at days 3–5 and days 7–10, and then weekly during the hospital stay. Additionally, patients were invited to 3- and 12-months follow-ups including blood sampling for biochemistry and biobanking. Serum was stored at − 80 °C, and thawed < 3 times. The ratio of partial pressure of oxygen in arterial blood (PaO2) to the fraction of inspiratory oxygen concentration (FiO2; P/F-ratio) was calculated for all patients. FiO2 in nonmechanically ventilated patients was approximated from supplementation of oxygen as found in the instructions for the EPIC II study [16]. The study was approved by the Regional Committee for Medical and Health Research Ethics in South-Eastern Norway (reference number 106624). All participants gave informed consent prior to inclusion, either directly or through a legally authorized representative.

Healthy control (HC) samples were obtained from healthy Norwegian blood donors at the Blood bank of Oslo University Hospital, under the general consent given by blood donors. The samples were collected in 2015 before the COVID-19 pandemic. Due to anonymity, there are no clinical or extended demographic data apart from age and gender available for the HC.

### Calprotectin and DNase

#### Calprotectin mixed monoclonal assay

The calprotectin ELISA is based on a mixture of monoclonal antibodies (ProMab Corp, Richmond, CA, USA) to ensure that all calprotectin in biological materials containing both histone and DNA fragments could be reliably assayed [17]. The mixed monoclonal (MiMo) antibodies, selected to react with all chromatography fractions of stool extracts from inflammatory bowel syndrome patients, were used both for the coating of microwells and preparation of a HRP conjugate. In brief, wells were coated with 5 µg/mL MiMo in 0.1 M sodium citrate at pH 6 for two hours at RT. After washing, standards (recombinant calprotectin) and samples were adequately diluted and incubated via shaking for 40 min at RT, followed by washing and incubation with HRP-anti-MiMo. The calprotectin levels were measured at 450 nm and expressed in ng/mL. The assay range is from 5 to 1000 ng/mL, with a coefficient of variation (CV) of about 5%.

#### Competitive DNase ELISA

In brief, a competitive DNase assay was established using chicken antibodies (NABAS, Ås, Norway) as a coat and recombinant human DNase (Roche, Basel, Switzerland) that was conjugated with HRP and mixed with a sample to be examined for readout. Readings at 450 nm after the addition of substrate were inversely proportional to the DNase concentration in the sample, expressed in ng/mL [18]. The assay range is from 5 to 500 ng/mL, with a CV of about 10%.

### Statistical analysis

Continuous normally distributed demographic variables were compared with Student’s t-test and presented as mean ± SD, whereas non-normally distributed variables were presented as median (25th/75th percentile) and compared with the Mann–Whitney U test. Categorical data were compared using the chi-square test.

For comparing the temporal profiles of groups during 10-day hospitalization, we utilized linear mixed models using untransformed levels and a log link function. Subject was used as random effect and time and outcome measure as fixed effects (also as interaction). Covariates used were age, sex, and any comorbidity. Type of oxygen supplementation was also tested as a covariate when assessing survival. HC were only assessed once and used for comparison with patients groups at various timepoints using a general linear model with calprotectin or DNase as dependent, group (i.e. ward, intensive care unit or high dependency unit (ICU/HDU), survivors and non-survivors; covid waves; long term follow-up samples at 3 months and 1 year vs. HC) as fixed, and age and sex as covariates.

For comparing admission levels of calprotectin, ferritin and CRP on ICU/HDU admission we first used ROC analysis and determined optimal cut-off using Youden’s index. Following dichotomization according to these cut-offs, markers association with ICU/HDU was further assessed using logistic regression with different models; (i) univariate, (ii) forward conditional regression with all three markers (iii) markers significant in ii were further adjusted with age, sex and any comorbidity and (iv) combination of dichotomized levels for two markers giving a parameter with three scales: L/L, below cutoff levels of both markers; H/L and L/H, i.e. high level of one marker and low level of the other; H/H, above cutoff level of both markers.

Simple correlations were assessed by Spearman. A stepwise multivariable regression analysis was used to identify predictors of baseline (i.e., < 48 h) calprotectin levels. Significant demographic and clinical parameters were included. Differences in wave demographics were compared by one-way ANOVA or Kuskal Wallis based on distribution, categorical differences were assessed with chi-square. Statistics were performed using SPSS version 29.0.0.0. A two-sided p-value of < 0.05 was deemed statistically significant.

## Results

### Baseline characteristics and outcomes of the study population

Demographic data and clinical characteristics according to clinical outcomes are shown in Table 1. A total of 252 patients were included for assessing calprotectin and DNase levels in ward patients (*n* = 174) and ICU/HDU patients (*n* = 78) during the first 10 days of hospitalization. For comparison, 17 (mean age ± SD: 52 ± 11, 8 female) and 10 (mean age ± SD: 53 ± 13, 5 female) HC were included for calprotectin and DNase analyses, respectively. Patients were an average of 56 (ward) and 60 (ICU/HDU) years old, predominantly male (61% and 65%, respectively). ICU/HDU patients had lower P/F ratio (Median P/F ratio ≤ 26.6 kPa (≤ 200 mmHg), which is regarded as the upper cutoff for moderate acute respiratory distress syndrome in the literature [19]), received oxygen therapy for a longer time and a greater proportion of patients received dexamethasone. Further, ICU/HDU patients had lower lymphocyte and higher neutrophil counts and generally higher levels of systemic inflammatory markers.

**Table 1:** Baseline characteristics in hospitalized COVID-19 patients according to ICU/HDU admission

	Ward (*n* = 174)	ICU/HDU (*n* = 78)
**Age, years,**	55.8 ± 16.3	59.7 ± 12.6*
Male sex, n (%)	106 (61)	51 (65)
BMI, kg/m^2^	28.2 ± 5	29.9 ± 5.2
Obesity (BMI ≥ 30), n (%)	40 (23)	29 (37)*
Ethnicity, % white/arabic/black/asian/other	63/13/8/11/4	57/10/10/19/5
Smoking, % former/current/unknown	29/6/8	35/5/13
Dexamethasone use n (%)	66 (38)	68 (87)***
Antiviral therapy, n (%)	2 (1)	2 (3)
Symptom duration, days	8.6 ± 5.5	8.3 ± 5.5
COVID-19 vaccination, % yes/unknown	8/8	3/8
COVID-19 wave, % 1/2/3	25/44/31	13/54/33*
Any oxygen therapy, n (%)	121 (70)	76 (97)***
Oxygen type, none/nasal/HFNC/ NIV/IMV/unknown, n (%)	53/121/0/0/0/0 (30/70/0/0/0/0)	1/9/10/23/34/1*** (1/12/13/29/44/1)
Oxygen therapy duration, days	5 ± 4.8	18.2 ± 13.5***
Bacterial pneumonia, n (%)	17 (10)	24 (31)***
P/F ratio, kPa	43.2 (34.8, 48.7)	24.6 (15.2, 35.6)***
Outcome		
60-day mortality, n (%)	6 (3)	23 (29)***
**Comorbidities**,** n (%)**		
Chronic cardiac disease	31 (18)	16 (21)
Hypertension	55 (32)	29 (37)
Chronic pulmonary disease	14 (8)	9 (12)
Asthma	31 (18)	16 (21)
Chronic kidney disease	17 (10)	6 (8)
Chronic neurological disorder	10 (6)	2 (3)
Cancer	6 (3)	3 (4)
Diabetes	41 (24)	17 (22)
Any comorbidity	105 (60)	51 (65)
**Laboratory analysis at admission**:		
Haemoglobin, g/dL	13.1 ± 1.8	12.8 ± 1.7
White blood cell count, × 10^9^/L	6.4 ± 3	7.6 ± 3.9**
Lymphocyte count, × 10^9^/L	1.15 ± 0.52	0.78 ± 0.39***
Neutrophil count, × 10^9^/L	4.8 ± 2.9	6.5 ± 3.7***
Creatinine, µM	86 ± 74	81 ± 33
C-reactive protein, mg/L	49 (21,104)	88 (43, 147)***
Ferritin, µg/L	515 (227, 897)	871 (455, 1552)***
D-dimer, mg/L	0.6 (0.4, 1)	1 (0.4, 1.9)**

Continuous data are given as mean ± SD or median (25th, 75th) percentile

Abbreviations: BMI, body mass index; Nasal, nasal oxygen cannula; HFNC, high-flow nasal cannula; NIV, non-invasive mechanical ventilation; IMV, invasive mechanical ventilation; P/F, ratio of partial pressure of oxygen in arterial blood (PaO2) to the fraction of inspiratory oxygen concentration (FiO2). ICU, intensive care unit; HDU, high dependency unit; COVID-wave (see methods for definition)

Continuous normally distributed demographic variables were compared with Student’s t-test, whereas non-normally distributed variables were compared with the Mann–Whitney U test. Categorical data were compared using the chi-square test

*p < 0.05, **p < 0.01, ***p < 0.001 vs. Ward

### Calprotectin and DNase during hospitalization

**Fig. 1:**
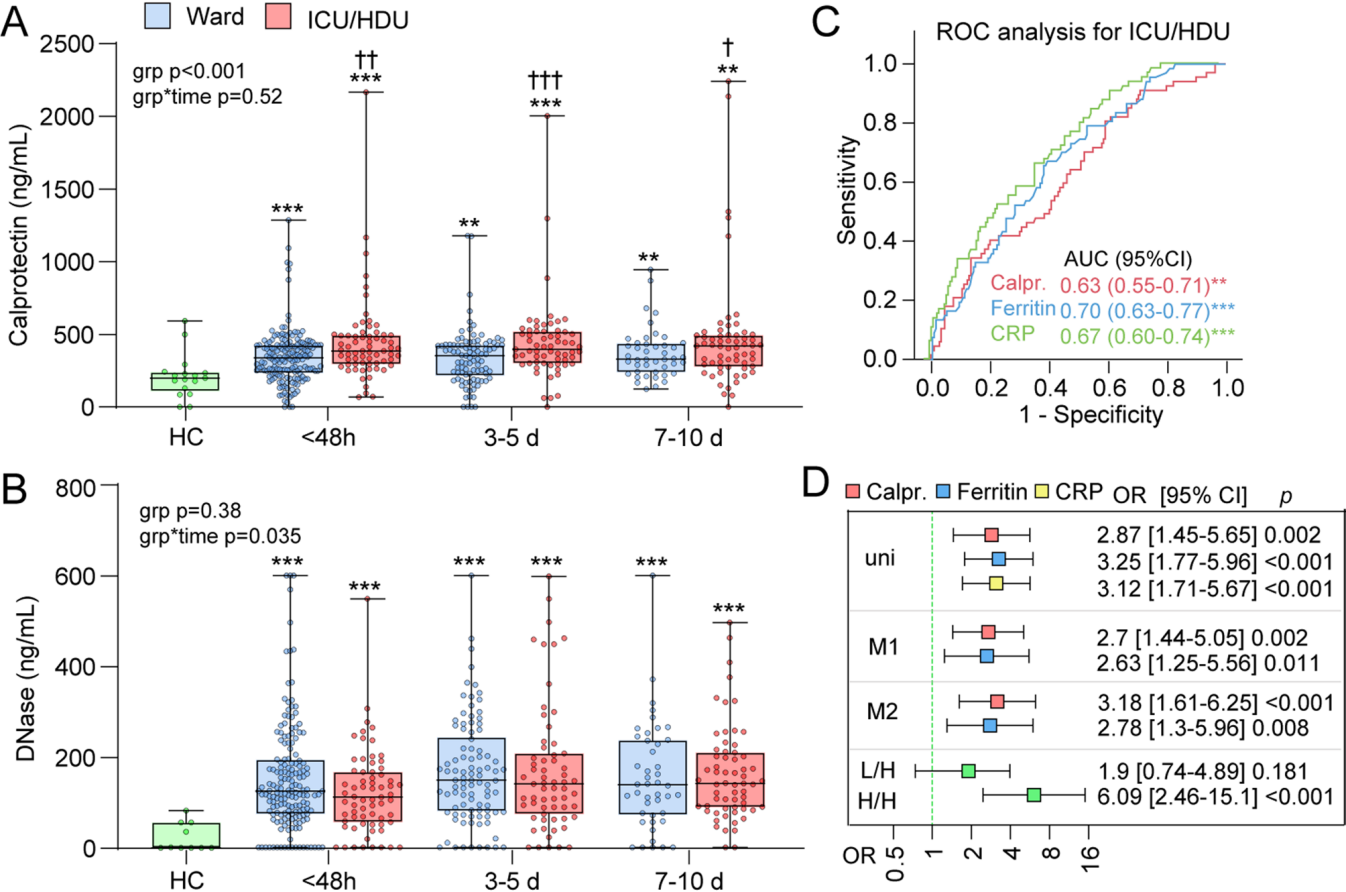
Temporal profile of calprotectin and deoxyribonuclease (DNase) according to intensive care unit (ICU)/high dependency unit (HDU) admission during the first 10 days of hospital admission and comparison with established biomarkers CRP and ferritin. Temporal profile of calprotectin (**A**) and deoxyribonuclease (DNase) (**B**). Groups were compared using linear mixed model with calprotectin or DNase as dependent, group as fixed and age, sex and presence of any comorbidity as covariates. The grp and grp*time p-values reflect the effect of ICU/HDU and ICU/HDU*time interaction from the linear mixed model analysis. **p < 0.01, ***p < 0.001 vs. Healthy controls (HC); ^†^*p* < 0.05, ^††^*p* < 0.01, ^†††^*p* < 0.001 vs. Ward. Of the 174 Ward and 78 ICU/HDU, not all patients had samples at all time-points. Numbers in each group at different time-points: Ward/ICU-HDU < 48 h: 168/67; 3-5d: 94/63; 7-10d: 44/62. For controls there were 17 samples for calprotectin and 10 for DNase. **(C)** Receiver operating curve (ROC) analysis showing area under the curve (AUC) for admission levels of calprotectin, CRP and ferritin in relation to ICU/HDU admission. (**D**) Logistic regression showing association (Odds ratio, OR) between dichotomized levels of markers based on Youden’s index determined from the ROC analysis in C. Cut-offs: calprotectin, 283 ng/mL; Ferritin, 628 µg/L; CRP, 58 mg/L. Uni, univariate model; M1, forward conditional regression; M2, adjustment with age, sex and any comorbidity; L/H, low level of one marker and high level of the other; H/H, above cutoff level of both markers (*n* = 82). Below cutoff levels of both markers were the reference group

ICU/HDU patients had consistently higher calprotectin levels and DNase levels at all timepoints compared to HC (Fig. 1A and B), and higher levels of calprotectin compared to ward patients (Fig. 1A). In contrast, there were no differences in DNase levels between ICU/HDU and ward patients at any time-point during hospitalization (Fig. 1B).

Next, we compared admission levels of calprotectin with CRP and ferritin in relation to ICU/HDU. As shown in Fig. 1C, these markers had modest but significant associations with ICU/HDU admittance (AUCs between 0.63 and 0.70). We next dichotomized levels using Youden’s index and assessed univariate associations with ICU/HDU with logistic regression (top of Fig. 1D). This analysis suggested that high levels (i.e. above cut-off) of these markers gave a 2.9 (Calprotectin), 3.1 (CRP) and 3.3 (ferritin) times higher odds of ICU/HDU admittance. Further, including all markers in a forward conditional regression indicated that calprotectin and ferritin gave independent information on prognosis (model 1, M1). Adjustment with age, sex and any comorbidity had no effect on this association (M2). Finally, we combined dichotomized calprotectin and ferritin giving a parameter with three scales: L/L, below cutoff levels of both markers; H/L and L/H, i.e. high level of one marker and low level of the other; H/H, above cutoff level of both markers. As shown at the bottom of the logistic regression in Fig. 1D, having high levels of both markers was associated with a 6.1 times higher odds of ICU/HDU admission.

### Predictors of calprotectin levels

**Fig. 2:**
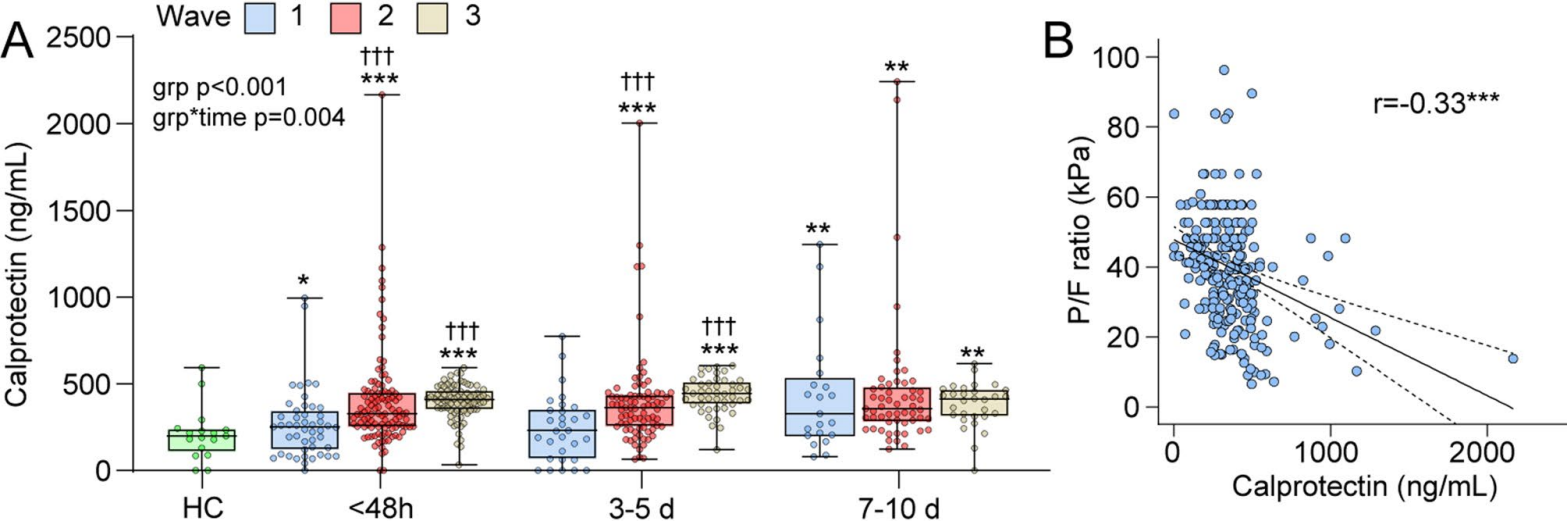
Clinical and demographic correlates to calprotectin.** (A)** Temporal profile of calprotectin according to COVID-wave (see material and methods for definition) during the first 10 days of hospital admission. Groups were compared using linear mixed model with calprotectin or DNase as dependent, group as fixed and age, sex and presence of any comorbidity as covariates. The grp and grp*time p-values reflect the effect of wave and wave*time interaction from the linear mixed model analysis. *p < 0.05, **p < 0.01, ***p < 0.001 vs. Healthy controls (HC); †††*p* < 0.001 vs. wave 1. Correlation analysis (Spearman) between calprotectin and **(B)** P/F ratio. ***p < 0.001

To determine predictors for baseline calprotectin and DNase levels we performed a linear regression analysis, presented in Supplemental Table 1 Male sex, COVID-19 wave and low P/F ratio were significant predictors of calprotectin levels and the focus for further correlation analysis (Fig. 2A-B). Demographics of patients according to waves are given in Supplemental Table 2 COVID-19 wave 2 and 3 had higher calprotectin levels than wave 1 at admission and 3–5 days after hospital admission (Fig. 2A). The negative correlation with P/F ratio is illustrated (Fig. 2B).

### Calprotectin and DNase according to survival

**Fig. 3:**
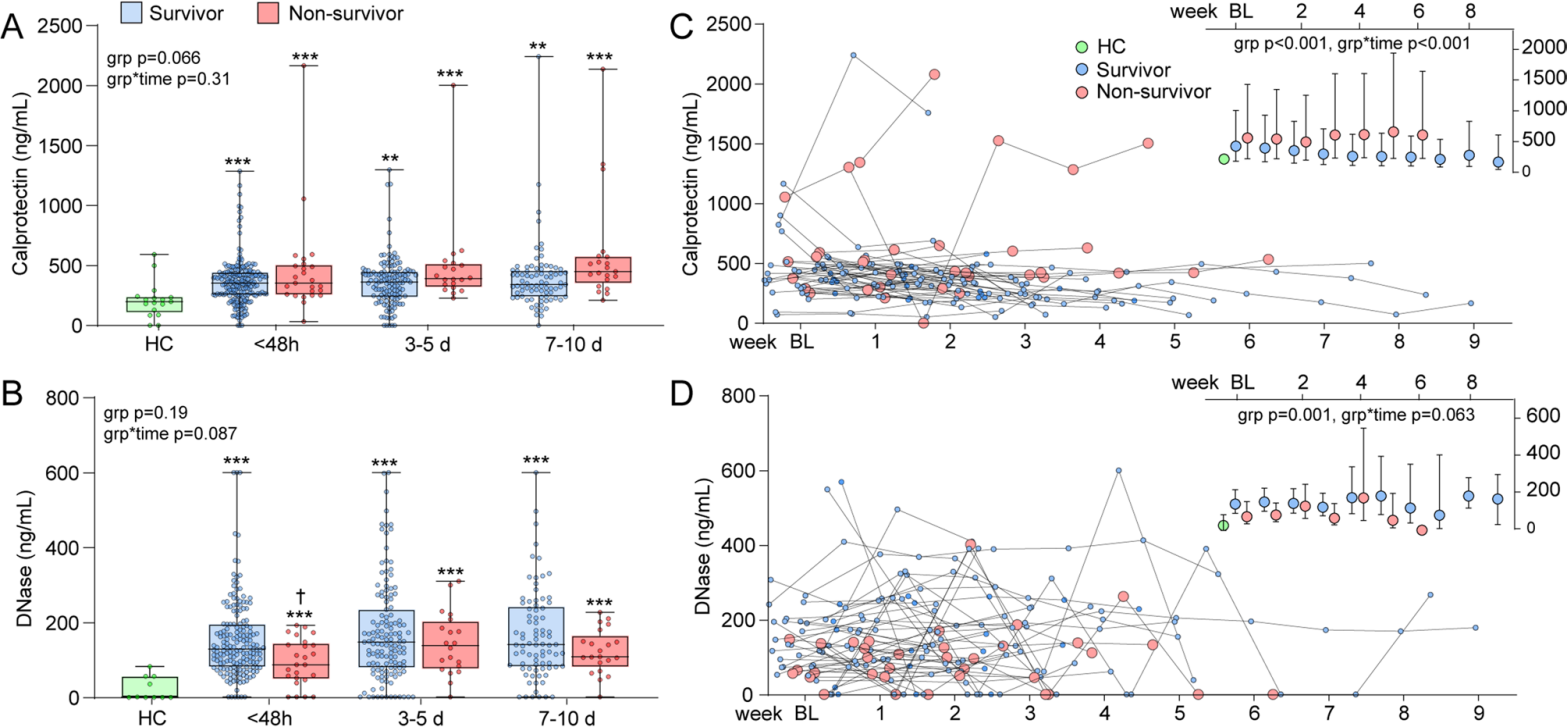
Temporal profile of calprotectin and deoxyribonuclease (DNase) according to survival. Boxplots of **(A)** Calprotectin and **(B)** DNase levels according to 60-day survival in survivors (*n* = 223) and non-survivors (*n* = 29) and compared to healthy controls (HC). Groups were compared using linear mixed models with calprotectin or DNase as dependent, group as fixed and age, sex and presence of any comorbidity as covariates. The grp and grp*time p-values reflect the effect of survival and survival*time interaction from the linear mixed model analysis. Not all patients had samples at all time-points. Numbers in each group at different time-points: Survivors/Non-survivors < 48 h: 211/25; 3-5d: 135/20; 7-10d: 81/21. For controls there were 17 samples for Calprotectin and 10 for DNase. **p < 0.01, ***p < 0.001 vs. HC; †*p* < 0.05 vs. Survivors. Weekly measurements of **(C)** calprotectin and **(D)** DNase levels in patients with extended stay according to 60-day survival in survivors (*n* = 39) and non-survivors (*n* = 9). The top right corner graph shows the linear mixed model analysis according to these groups as estimated marginal means with 95% confidence intervals using age, sex and comorbidities as covariates. The grp and grp*time p-values reflect the overall group difference and interaction with time from the linear mixed model analysis

Of the 252 patients included in the study, 29 died during the first 60 days. Demographics in survivors and non-survivors is shown in Supplemental Table 3. Non-survivors had similar demographics as ICU/HDU patients (Table 1) and in addition, were older, more males and had more comorbidities than survivors. No differences in calprotectin levels according to survival was detected during the initial hospitalization (Fig. 3A) while DNase was lower only at baseline (Fig. 3B), compared to survivors. The difference at baseline remained significant following additional adjustment with type of oxygen supplementation (*p* = 0.007).

Weekly measurements of calprotectin and DNase were available in 39 survivors and 9 non-survivors with extended stay up to 9 weeks. Demographics of this population is shown in Supplemental Table3. As shown in Fig. 3C and D, calprotectin levels remained higher and DNase lower in non-survivors during this longer follow-up. Further adjustment with type of oxygen supplementation had no large impact on calprotectin (grp *p* = 0.003, grp*time *p* < 0.001) or DNase (grp *p* < 0.001, grp*time *p* = 0.054).

### Calprotectin and DNase at long-term follow-up

**Fig. 4:**
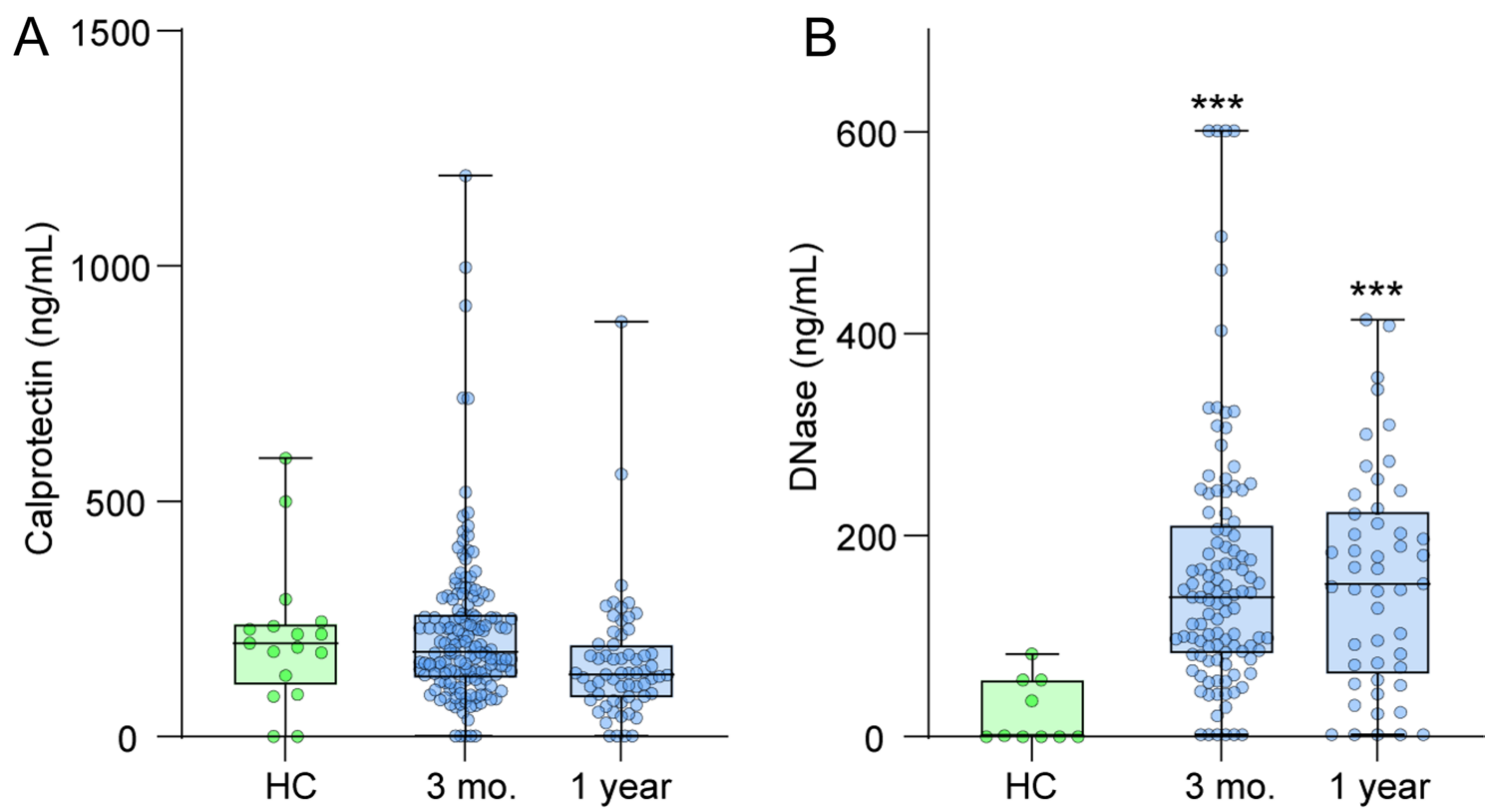
Boxplots of **(A)** calprotectin and **(B)** deoxyribonuclease (DNase) levels at 3 months and 1 year follow-up. Patients and healthy controls (HC) were compared at each timepoints with controls using general linear model with calprotectin and DNase as dependent, group as fixed and age and sex as covariates. ***p < 0.001 vs. HC

We had available samples from 158 patients at 3 months and 62 patients at 1 year. Demographics of these patients are shown in Supplemental Table 4. As shown in Fig. 4, no differences in calprotectin levels compared to HC were found, but DNase level were markedly higher in patients at both time-points.

## Discussion

The present study demonstrates the dynamic changes of calprotectin and DNase in hospitalized COVID-19 patients up to 1 year of follow-up. Key findings were: (i) ICU/HDU patients had consistently higher calprotectin and DNase levels during hospitalization compared to HC, and higher calprotectin levels compared to ward patients; (ii) adding calprotectin to ferritin improved the prognostic accuracy for admission to ICU/HDU (iii) high calprotectin levels at admission were associated with later COVID-19 waves and low P/F ratio; (iv) non-survivors displayed high calprotectin levels during extended assessment in patients with prolonged hospital stay; (v) DNase levels were similar in survivors and non-survivors during hospitalization but were lower within < 48 h of admission and during extended hospital stay in non-survivors; (vi) Finally, DNase levels were higher in COVID-19 patients than in HC at 3 months and 1-year follow-up.

Similar to our findings, several reports with longitudinal measurements of calprotectin have demonstrated an increase in circulating calprotectin in hospitalized COVID-19 patients compared to HC and higher levels in hospitalized non-survivors and ICU/HDU COVID-19 patients. In the first report with serial measurements of circulating calprotectin in hospitalized patients, calprotectin increased when oxygenation worsened and was higher in patients who required mechanical ventilation as compared to those who remained free of intubation [14]. Another study with multiple measurements during hospital admission and > 90 days after hospital admission showed increased levels of calprotectin during the hospital admission significantly related to mortality status and ICU admission during mainly the first wave of COVID-19 (March to August 2020; [12]). Chapuis et al. introduced an algorithm based on serial measurements of calprotectin up to 30 days since diagnosis to monitor the probability of a poor outcome in individual patients with moderate COVID-19 at admission [10]. Another study has also shown that circulating calprotectin levels increased in non-survivors and did not significantly evolve over time in survivors during ICU hospitalization [13]. Increasing calprotectin levels from admission to day 7 has been shown in a multicenter ICU study to be independently associated with higher mortality at 12 months [11]. A recently published study with serial measurements of circulating calprotectin in hospitalized COVID-19 patients concluded that levels on admission and their subsequent dynamic alterations until discharge or intubation/death could predict the occurrence of severe respiratory failure and mortality [1]. Notably, our finding that calprotectin levels were augmented in the later COVID-19 waves is consistent with another study showing higher neutrophil counts in later waves, suggesting variability of the various virus variants in triggering immune responses [20]. There could be other reasons than viral strains, but vaccination or earlier exposure to SARS-CoV-2 could also explain an increased immune response in later waves. We also found that oxygen therapy and the differentiation in oxygen type was not different between the different waves, but dexamethasone use increased in the two last waves compared to the first wave (Supplemental Table 2) as expected regards to the study findings in the Recovery trial indicating better survival with dexamethasone use in patients receiving any oxygen therapy [21].

Only two studies have investigated the levels of calprotectin > 90 days after hospital admission. In support of our findings showing normalization at 3-month and 12 months follow up, Mellet et al. reported a substantial drop in calprotectin at 90 days follow-up compared to measurements done between hospital admission and 28 days after admission [12]. In another study measuring circulating calprotectin in ICU and non-ICU patients, only once at four months after the acute phase of COVID-19, they found significantly higher levels of calprotectin in ICU-patients, and they found a negative correlation between calprotectin levels and diffusing capacity for carbon monoxide or forced vital capacity [22].

A recent study reported low DNase in ICU patients at admission with normalization at day 6–8 and increased levels at the time of discharge [23]. Although we found no difference in DNase levels between ICU and ward patients, lower DNase levels within 48 h of hospital admission were found in non-survivors compared to survivors. DNase treatment in a murine model of COVID-19 decreased levels of NETs, TNF-α and IL-6, improved disease progression and reduced lung, heart and kidney injuries [24]. Similar findings have been found in an experimental murine model of sepsis induced by cecal ligation and puncture where DNase administration reduced NETs and IL-6 levels, suppressed organ damage and bacterial dissemination, and decreased mortality [25].

We recently reported prolonged and consistently elevated complement activation during long-term follow-up in hospitalized COVID-19 patients [26]. To our knowledge, this is the first report demonstrating a similar consistent and prolonged elevation of DNase following SARS-CoV-2 infection. We speculate that this long-lasting activation may be related to long-COVID, but could also be explained by differences among patient populations and lack of clinical data makes it impossible to align this prolonged activation of DNase to long COVID. Further investigations should be done to explore if a prolonged DNase activation in up to one year or more is also found in other community-acquired pneumonias (e.g. influenza and others). To our knowledge this has neither been observed before.

The strength of this study lies in its simultaneous temporal assessment of calprotectin and DNase levels during hospitalization and throughout long-term follow-up in patients with COVID-19. In addition, the relatively large, multicenter cohort spans multiple pandemic waves, reflecting the extended duration of data collection and enhancing the study’s generalizability. Nonetheless, several limitations should be acknowledged. These include a small control group with only healthy individuals despite being age- and sex-matched. Additionally, a non-hospitalized control group with similar comorbidities as the included COVID-19 patients could have added more strength to the study. Finally, attrition and survivorship bias in follow-up and no functional status or no recordings of long covid manifestations also represents limitations to our study.

## Conclusions

COVID-19 non-survivors had higher levels of calprotectin during prolonged hospitalization compared with survivors. High calprotectin at admission was related to later pandemic waves and impaired oxygenation. Adding calprotectin to ferritin improved the prognostic accuracy for admission to ICU/HDU. DNase was lower at admission in non-survivors compared to survivors. Additionally, DNase was increased during hospital admission extending to 1-year follow-up compared to HC indicating a potential role for DNase in long COVID.

## Supplementary Information

Below is the link to the electronic supplementary material.

Supplementary Material 1

## Data Availability

The datasets used and generated in this study are included in this published article and its supplementary file.

## Abbreviations

DNase: Deoxyribonuclease
ICU: Intensive care unit
HC: Healthy controls
HDU: High dependency unit
ISARIC: International Severe Acute Respiratory and Emerging Infection Consortium
MiMo: Mixed Monoclonal
NETs: Neutrophil Extracellular Traps
P/F: PaO2/FiO2
