# Supplementary material for: Serial blood serum measurements of calprotectin and deoxyribonuclease in COVID-19 patients during hospitalization and recovery until one year: a prospective, multicenter, observational study
Source: BMC Infect Dis. 2026 Mar 2;26:723. doi: 10.1186/s12879-026-12939-x (PMC13059574; doi:10.1186/s12879-026-12939-x)
Supplement: Supplementary file 1 — Supplementary Material 1 [file 12879_2026_12939_MOESM1_ESM.docx]

**Supplemental Table 1.** Baseline correlations between Calprotectin, DNase and clinical variables.

|  | Calprotectin | | | | DNase | | |
| --- | --- | --- | --- | --- | --- | --- | --- |
|  | All | Multi | Ward | ICU/HDU | All | Ward | ICU/HDU |
| Calprotectin | 1.00 |  | 1.00 | 1.00 | 0.07 | -0.01 | 0.12 |
| DNase | 0.07 |  | 0.12 | -0.01 | 1.00 | 1.00 | 1.00 |
| Oxygen therapy | 0.31*** |  | 0.33*** | 0.05 | 0.12 | -0.10 | 0.16* |
| Male sex | 0.24*** | 0.18* | 0.29*** | 0.11 | 0.02 | 0.04 | 0.03 |
| Age | -0.04 |  | -0.04 | -0.11 | -0.08 | -0.11 | -0.05 |
| Symptom duration | 0.16* |  | .16* | 0.19 | -0.04 | -0.11 | -0.03 |
| COVID-19 wave | 0.36*** | 0.32*** | .38*** | 0.28* | -0.01 | -0.01 | 0.01 |
| P/F ratio | -0.33*** | -0.27*** | -0.27*** | -0.41*** | 0.05 | 0.24* | -0.07 |
| BMI | 0.15 |  | 0.13 | 0.14 | 0.07 | 0.09 | 0.07 |
| Chronic cardiac disease | -0.11 |  | -0.11 | -0.13 | -0.02 | -0.11 | 0.01 |
| Hypertension | 0.03 |  | -0.01 | 0.09 | 0.06 | 0.07 | 0.06 |
| Chronic pulmonary disease | -0.01 |  | -0.01 | -0.02 | 0.07 | -0.07 | 0.16* |
| Asthma | 0.03 |  | 0.03 | -0.02 | 0.01 | -0.03 | 0.03 |
| Chronic kidney disease | 0.00 |  | 0.05 | -0.07 | 0.01 | 0.11 | -0.02 |
| Chronic neurological disorder | 0.01 |  | 0.05 | 0.00 | -0.03 | -0.06 | -0.04 |
| Cancer | -0.09 |  | -0.11 | -0.10 | -0.19** | -0.26* | -0.16* |
| Diabetes Mellitus | -0.04 |  | -0.05 | 0.02 | -0.10 | -0.21 | -0.07 |
| Haemoglobin | 0.06 |  | .158* | -0.16 | 0.12 | 0.21 | 0.08 |
| White blood cell count | 0.36*** |  | 0.40*** | 0.27* | -0.02 | -0.17 | 0.07 |
| Lymphocyte count | -0.19** |  | -0.15 | -0.01 | 0.05 | -0.04 | 0.04 |
| Neutrophil count | 0.43*** |  | 0.46*** | 0.31* | -0.02 | -0.16 | 0.07 |
| Platelet count | 0.11 |  | 0.13 | 0.09 | -0.02 | 0.00 | -0.02 |
| CRP | 0.39*** |  | 0.49*** | 0.02 | -0.02 | 0.06 | -0.05 |
| Ferritin | 0.42*** | 0.13 | 0.41** | 0.27* | -0.02 | -0.07 | 0.01 |
| D-dimer | 0.12 |  | 0.09 | 0.12 | -0.02 | -0.18 | 0.01 |
| Bacterial pneumonia | -0.15* |  | -0.24** | 0.04 | -0.02 | -0.09 | -0.19* |

Abbreviations: BMI, body mass index; CRP, C-reactive protein; DNase, deoxyribonuclease; P/F, ratio of partial pressure of oxygen in arterial blood (PaO2) to the fraction of inspiratory oxygen concentration (FiO2). COVID-wave (see methods for definition).

Correlations were assessed by Spearman.

*p<0.05, **p<0.01, ***p<0.001

**Supplemental Table 2.** Baseline characteristics in different waves of hospitalized COVID-19 patients

|  | Wave 1 (n=53) | Wave 2 (n=119) | Wave 3 (n=80) | P-value |
| --- | --- | --- | --- | --- |
| Age, years | 59.7±15.5 | 58.9±15.4 | 52.4±14.4 | 0.004 |
| Male sex, n (%) | 37 (70) | 68 (57) | 52 (65) | 0.24 |
| BMI, kg/m^2^ | 27.6±5.5 | 28.7±5.1 | 29.1±4.9 | 0.46 |
| Obesity (BMI≥30), n (%) | 7 (13) | 39 (33) | 23 (29) | 0.028 |
| Ethnicity, % white/arabic/black/asian/other | 66/6/15/11/2 | 56/12/8/16/8 | 66/16/4/12/3 | 0.14 |
| Smoking, % former/current/unknown | 21/6/2 | 38/5/10 | 28/8/14 | 0.036 |
| Dexamethasone use n (%) | 4 (8) | 81 (68) | 49 (61) | <0.001 |
| Antiviral therapy, n (%) | 0 (0) | 3 (3) | 1 (1) | 0.44 |
| Symptom duration, days | 10.3±6.6 | 8.3±5.9 | 7.8±3.6 | 0.028 |
| COVID-19 vaccination, % yes/unknown | 0/0 | 0/3 | 9/10 | 0.19 |
| Any oxygen therapy, n (%) | 38 (72) | 97 (82) | 62 (78) | 0.23 |
| Oxygen type, none/nasal/HFNC/ NIV/IMV/unknown, n (%) | 15/29/2/0/7/0  (28/55/4/0/13/0) | 22/62/5/14/16/0  (19/52/4/12/13/0) | 17/39/3/9/11/1  (21/49/4/11/14/1) | 0.42 |
| Oxygen therapy duration, days | 8.1±8.1 | 10.8±11.7 | 10.2±12.1 | 0.46 |
| Bacterial pneumonia, n (%) | 7 (13) | 22 (18) | 12 (15) | 0.48 |
| P/F ratio, kPa | 28.1 (17.0, 37.8) | 39.6 (27.8, 48.2) | 38.2 (25.2, 43.2) | 0.065 |
| Outcome |  |  |  |  |
| ICU/HDU, n (%) | 10 (19) | 42 (35) | 26 (33) | 0.093 |
| 60-day mortality, n (%) | 6 (11) | 17 (14) | 6 (8) | 0.34 |
| **Comorbidities, n (%)** |  |  |  |  |
| Chronic cardiac disease | 14 (26) | 23 (19) | 10 (13) | 0.13 |
| Hypertension | 17 (32) | 53 (45) | 14 (18) | <0.001 |
| Chronic pulmonary disease | 2 (4) | 16 (13) | 5 (6) | 0.070 |
| Asthma | 12 (23) | 18 (15) | 17 (21) | 0.57 |
| Chronic kidney disease | 5 (9) | 13 (11) | 5 (6) | 0.53 |
| Chronic neurological disorder | 3 (6) | 4 (3) | 5 (6) | 0.61 |
| Cancer | 1 (2) | 5 (4) | 3 (4) | 0.75 |
| Diabetes | 7 (13) | 34 (29) | 17 (21) | 0.34 |
| Any comorbidity, n (%) | 30 (57) | 81 (68) | 45 (56) | 0.163 |
| **Laboratory analysis at admission:** |  |  |  |  |
| Haemoglobin, g/dL | 13.3±1.9 | 12.6±1.7 | 13.4±1.6 | 0.007 |
| White blood cell count, × 10^9^/L, | 6.6±3.4 | 7.1±3.6 | 13.4±1.6 | 0.36 |
| Lymphocyte count, × 10^9^/L | 1.11±0.44 | 1.02±0.53 | 1.02±0.55 | 0.48 |
| Neutrophil count, × 10^9^/L | 5.0±3.4 | 5.6±3.5 | 5.0±2.9 | 0.29 |
| Creatinine, µM | 89±54 | 85±68 | 80±64 | 0.75 |
| C-reactive protein, mg/L | 64 (20, 139) | 56 (25, 111) | 55 (28, 115) | 0.93 |
| Ferritin, µg/L | 639 (242, 1112) | 600 (332, 1164) | 604 (290, 1054) | 0.7 |
| D-dimer, mg/L | 0.60 (0.37, 1.60) | 0.80 (0.43, 1.60) | 0.60 (0.30, 0.90) | 0.083 |

Continuous data are given as mean ± SD or median (25th, 75th) percentile.

Abbreviations: BMI, body mass index; Nasal, nasal oxygen cannula; HFNC, high-flow nasal cannula; NIV, non-invasive mechanical ventilation; IMV, invasive mechanical ventilation; ICU, intensive care unit; HDU, high dependency unit.

Differences in wave demographics were compared by one-way ANOVA or Kuskal Wallis based on distribution, categorical differences were assessed with chi-square.

**Supplemental Table 3.** Baseline characteristics in hospitalized COVID-19 patients according to 60-day mortality

|  | Survivors (n=223) | Non-survivors (n=29) |
| --- | --- | --- |
| Age, years | 55.1±14.8 | 71.2±11.7*** |
| Male sex, n (%) | 134 (60) | 23 (79)* |
| BMI, kg/m^2^ | 28.9±5.1 | 27.1±4.7 |
| Obesity (BMI≥30), n (%) | 63 (28) | 6 (21) |
| Ethnicity, % white/arabic/black/asian/other | 61/13/9/13/4 | 63/4/0/22/11* |
| Smoking, % former/current | 28/6 | 52/3* |
| Dexamethasone use n (%) | 112 (50) | 22 (76)** |
| Antiviral therapy, n (%) | 4 (2) | 0 (0) |
| Symptom duration, days | 8.7±5.7 | 6.9±3.6* |
| COVID-19 vaccination, % yes/unknown | 6/8 | 8/8 |
| COVID-19 wave, % 1/2/3 | 21/46/33 | 21/59/21 |
| Any oxygen therapy, n (%) | 171 (77) | 26 (90) |
| Oxygen type, none/nasal/HFNC/ NIV/IMV/unknown, n (%) | 51/125/9/18/19/1  (23/56/4/8/9/0.5) | 3/5/1/5/15/0***  (10/17/3/17/52/0) |
| Oxygen therapy duration, days | 8.9±10 | 18±14.9*** |
| Bacterial pneumonia, n (%) | 33 (15) | 8 (28)*** |
| P/F ratio, kPa | 40.0 (29.5, 48,3) | 18.5 (12.1, 35.1)*** |
| Outcome |  |  |
| ICU/HDU, n (%) | 55 (25) | 23 (79)*** |
| **Comorbidities, n (%)** |  |  |
| Chronic cardiac disease | 31 (14) | 16 (55)*** |
| Hypertension | 74 (33) | 10 (34) |
| Chronic pulmonary disease | 14 (6) | 9 (31)*** |
| Asthma | 42 (19) | 5 (17) |
| Chronic kidney disease | 16 (7) | 7 (24)** |
| Chronic neurological disorder | 10 (4) | 2 (7) |
| Cancer | 6 (3) | 3 (10) |
| Diabetes | 51 (23) | 7 (24) |
| Any comorbidity | 132 (59) | 24 (83)* |
| **Laboratory analysis at admission:** |  |  |
| Haemoglobin, g/dL | 13.1±1.7 | 12.3±1.8* |
| WBC, × 10^9^/L | 6.8±3.4 | 6.5±3.2 |
| Lymphocyte count, × 10^9^/L | 1.09±0.51 | 0.62±0.31*** |
| Neutrophil count, × 10^9^/L | 5.3±3.3 | 5.4±2.9 |
| Creatinine, µM | 80±52 | 119±118*** |
| C-reactive protein, mg/L | 53 (25, 115) | 83 (48, 137) |
| Ferritin, µg/L | 579 (269, 1090) | 832 (520, 1439)* |
| D-dimer, mg/L | 0.6 (0.4, 1.1) | 1.6 (0.8, 1.9)*** |

Continuous data are given as mean ± SD or median (25th, 75th) percentile.

Abbreviations: BMI, body mass index; Nasal, nasal oxygen cannula; HFNC, high-flow nasal cannula; NIV, non-invasive mechanical ventilation; IMV, invasive mechanical ventilation; P/F, ratio of partial pressure of oxygen in arterial blood (PaO2) to the fraction of inspiratory oxygen concentration (FiO2). ICU, intensive care unit; HDU, high dependency unit; COVID-wave (see methods for definition).

Continuous normally distributed demographic variables were compared with Student's t-test, whereas non-normally distributed variables were compared with the Mann–Whitney U test. Categorical data were compared using the chi-square test.

*p<0.05, **p<0.01, ***p<0.001 vs*.* Survivors.

**Supplemental Table 4.** Baseline characteristics in different subpopulations of hospitalized COVID-19 patients with extended sampling.

|  | weekly samples (n=48) | 3-month (n=158) | 1-year (n=62) |
| --- | --- | --- | --- |
| Age, years | 58.5±11.5 | 54.8±13.8 | 56.3±12.2 |
| Male sex, n (%) | 32 (67) | 94 (59) | 42 (68) |
| BMI, kg/m^2^ | 29.1±4.5 | 29.2±5.2 | 29.9±5.2 |
| Obesity (BMI≥30), n (%) | 14 (29) | 47 (30) | 11 (18) |
| Ethnicity, % white/arabic/black/asian/other | 53/13/11/19/4 | 64/10/10/12/4 | 71/8/8/11/2 |
| Smoking, % former/current/unknown | 31/8/8 | 33/5/5 | 34/7/0 |
| Dexamethasone use n (%) | 40 (83) | 73 (46) | 6 (10) |
| Antiviral therapy, n (%) | 2 (4) | 2 (1) | 0 (0) |
| Oxygen type, none/nasal/HFNC/ NIV/IMV/unknown, n (%) | 0/14/3/11/20/0  (0/29/6/23/42/0) | 36/89/4/15/14/0  (23/56/3/9/9/0) | 12/39/3/3/5/0  (19/63/5/5/8/0) |
| Symptom duration, days | 8.6±6.5 | 9.9±5.9 | 10.9±6.4 |
| COVID-19 vaccination, % yes/unknown | 8/4 | 1/1 | 0/0 |
| COVID-19 wave, % 1/2/3 | 15/58/27 | 33/47/20 | 97/3/0 |
| Any oxygen therapy, n (%) | 48 (100) | 122 (77) | 50 (81) |
| Oxygen therapy duration, days | 20.7±14.3 | 7.8±7.7 | 7.5±5.9 |
| Bacterial pneumonia, n (%) | 14 (30) | 19 (12) | 10 (16) |
| P/F ratio, kPa | 28.1 (17.0, 37.8) | 39.5 (28.1, 48.3) | 38.2 (25.2, 43.2) |
| Outcome |  |  |  |
| ICU/HDU, n (%) | 36 (75) | 37 (23) | 10 (16) |
| **Comorbidities, n (%)** |  |  |  |
| Chronic cardiac disease | 8 (17) | 24 (15) | 12 (19) |
| Hypertension | 17 (35) | 54 (34) | 22 (35) |
| Chronic pulmonary disease | 8 (17) | 10 (6) | 3 (5) |
| Asthma | 11 (23) | 32 (20) | 11 (18) |
| Chronic kidney disease | 5 (10) | 10 (6) | 1 (2) |
| Chronic neurological disorder | 1 (2) | 3 (2) | 0 (0) |
| Cancer | 2 (4) | 4 (3) | 1 (2) |
| Diabetes | 11 (23) | 33 (21) | 7 (11) |
| Any comorbidity | 33 (69) | 95 (60) | 34 (55) |
| **Laboratory analysis at admission:** |  |  |  |
| Haemoglobin, g/dL, | 12.8±2 | 13.2±1.6 | 13.4±1.4 |
| White blood cell count, × 10^9^/L, | 7.1±3.6 | 7.4±9.2 | 6.9±3.4 |
| Lymphocyte count, × 10^9^/L | 0.82±0.4 | 1.12±0.52 | 1.15±0.46 |
| Neutrophil count, × 10^9^/L | 5.9±3.4 | 5.2±3.3 | 5.3±3.3 |
| Creatinine, µM | 83±37 | 79±32 | 78±19 |
| C-reactive protein, mg/L | 85 (43, 145) | 51 (21, 114) | 75 (29, 152) |
| Ferritin, µg/L | 762 (411, 1415) | 572 (268, 1183) | 662 (318, 1218) |
| D-dimer, mg/L | 0.91 (0.46, 1.7) | 0.7 (0.4, 1.2) | 0.8 (0. 5, 1.6) |

Continuous data are given as mean ± SD or median (25th, 75th) percentile.

Abbreviations: BMI, body mass index; Nasal, nasal oxygen cannula; HFNC, high-flow nasal cannula; NIV, non-invasive mechanical ventilation; IMV, invasive mechanical ventilation; P/F, ratio of partial pressure of oxygen in arterial blood (PaO2) to the fraction of inspiratory oxygen concentration (FiO2). ICU, intensive care unit; HDU, high-dependency unit; COVID-wave (see methods for definition).
